# Proposal and validation of a new approach in tele-rehabilitation with 3D human posture estimation: a randomized controlled trial in older individuals with sarcopenia

**DOI:** 10.1186/s12877-024-05188-7

**Published:** 2024-07-08

**Authors:** Shichun He, Deyu Meng, Meiqi Wei, Hongzhi Guo, Guang Yang, Ziheng Wang

**Affiliations:** 1 Chinese Center of Exercise Epidemiology, Northeast Normal University, Renmin Street, Changchun, 130024 Jilin China; 2 AI Group, Intelligent Lancet LLC, Sacramento, CA 95816 USA; 3 Graduate School of Human Sciences, Waseda University, Tokorozawa, 3591192 Aitama Japan; 4 Advanced Research Center for Human Sciences, Waseda University, Tokorozawa, 3591192 Aitama Japan

**Keywords:** sarcopenia, Remote rehabilitation, Human pose estimation technology, Tai chi

## Abstract

**Objective:**

Through a randomized controlled trial on older adults with sarcopenia, this study compared the training effects of an AI-based remote training group using deep learning-based 3D human pose estimation technology with those of a face-to-face traditional training group and a general remote training group.

**Methods:**

Seventy five older adults with sarcopenia aged 60–75 from community organizations in Changchun city were randomly divided into a face-to-face traditional training group (TRHG), a general remote training group (GTHG), and an AI-based remote training group (AITHG). All groups underwent a 3-month program consisting of 24-form Taichi exercises, with a frequency of 3 sessions per week and each session lasting 40 min. The participants underwent Appendicular Skeletal Muscle Mass Index (ASMI), grip strength, 6-meter walking pace, Timed Up and Go test (TUGT), and quality of life score (QoL) tests before the experiment, during the mid-term, and after the experiment. This study used SPSS26.0 software to perform one-way ANOVA and repeated measures ANOVA tests to compare the differences among the three groups. A significance level of *p* < 0.05 was defined as having significant difference, while *p* < 0.01 was defined as having a highly significant difference.

**Results:**

(1) The comparison between the mid-term and pre-term indicators showed that TRHG experienced significant improvements in ASMI, 6-meter walking pace, and QoL (*p* < 0.01), and a significant improvement in TUGT timing test (*p* < 0.05); GTHG experienced extremely significant improvements in 6-meter walking pace and QoL (*p* < 0.01); AITHG experienced extremely significant improvements in ASMI, 6-meter walking pace, and QoL (*p* < 0.01), and a significant improvement in TUGT timing test (*p* < 0.05). (2) The comparison between the post-term and pre-term indicators showed that TRHG experienced extremely significant improvements in TUGT timing test (*p* < 0.01); GTHG experienced significant improvements in ASMI and TUGT timing test (*p* < 0.05); and AITHG experienced extremely significant improvements in TUGT timing test (*p* < 0.01). (3) During the mid-term, there was no significant difference among the groups in all tests (*p* > 0.05). The same was in post-term tests (*p* > 0.05).

**Conclusion:**

Compared to the pre-experiment, there was no significant difference at the post- experiment in the recovery effects on the muscle quality, physical activity ability, and life quality of patients with sarcopenia between the AI-based remote training group and the face-to-face traditional training group. 3D pose estimation is equally as effective as traditional rehabilitation methods in enhancing muscle quality, functionality and life quality in older adults with sarcopenia.

**Trial registration:**

The trial was registered in ClinicalTrials.gov (NCT05767710).

## Introduction

Sarcopenia is a debilitating age-associated condition characterized by the gradual and widespread degeneration of skeletal muscle mass and function [1], affecting 5–13% of adults aged 60–70 years and 11–50% of those aged 80 years or older worldwide [2]. Guidelines of the Asian Working Group for sarcopenia [3], the task force of the International Conference on sarcopenia and Frailty Research [4], and the Singapore Society for Geriatric Medicine [5] all strongly endorse exercise training as the first-line therapy for the management of sarcopenia. Exercise programs, in particular, represent a crucial component of rehabilitation and have been shown to have a favorable impact on sarcopenia [6–11], the implementation of Chinese traditional exercise resulted in a 27.8% reverse in sarcopenia [12]. Despite the known benefits, participation in and adherence to exercise training among older adults with chronic musculoskeletal conditions are even lower than among the general old population [13, 14]. Factors hindering older adults’ adherence to exercise include several aspects. Firstly, lack of resources, such as access to exercise facilities, equipment, but mainly professional coaches or doctors. Study shows most doctors have limited knowledge of sarcopenia [15]. And adequate screening and diagnosis is almost nonexistent in current clinical practice, which hampers interventions [16]. Secondly, logistical and psychological barriers, such as transportation and expense issues [13, 17]. The expenses associated with rehabilitation remain substantial, requiring extended periods of engagement with a medical practitioner or certified trainer [18]. Additionally, even when financially feasible, individuals may encounter limited access to medical resources within certain regions or nations [13, 19, 20]. Hence, effective and widely applicable rehabilitation therapy for sarcopenia has emerged as a critical issue.

Fortunately, tele-rehabilitation, the delivery of rehabilitation services at a distance by electronic information and communication technologies [21, 22], has been shown to provide comparable effects with in-person rehabilitation or better than no rehabilitation in various conditions [23]. Specifically, tele-rehabilitation has been demonstrated to enhance physical function [24], increase muscle strength [25], and improve the overall QoL [26] among sarcopenia patients. However, despite the potential of existing approaches to overcome location-related challenges, it is still necessary for prolonged professional supervision of the rehabilitation process for older adults with sarcopenia. The research suggests that incorrect exercise postures can result in temporary or permanent disabilities [27]. Therefore, a pressing question arises: how can remote rehabilitation for individuals with sarcopenia be conducted both accurately and effectively?

The rapid advancements in the field of artificial intelligence (AI) have opened up new avenues for tele-rehabilitation: integrating AI as a professional supervisory role to assist older adults with sarcopenia. Pose estimation, involves detecting the position and orientation of a person or an object from videos or images, which can enhance the accuracy [28] and quality [29] of tele-rehabilitation services based on 2D estimation. Due to the capability of 2D pose estimation to facilitate the measurement of changes in patients’ postures and movements, it can assist in assessing the function of their limbs or joints and tracking their recovery progress [30–32] and provide feedback during therapy sessions, which enables patients to understand and correct any posture or movement issues [29, 33]. However, to the best of our knowledge, previous studies have utilized 2D pose estimation, which may lose significant details, particularly in complex rehabilitation actions such as those in Chinese traditional exercise. In contrast, 3D pose estimation involves the estimation of the 3D orientation and position of joints (such as shoulders, elbows, wrists, hips, knees, and ankles) in human pose estimation. Thus, we proposed combining 3D pose estimation as a professional supervisory role with tele-rehabilitation exercise training to form a tele-rehabilitation program.

In summary, we propose a novel approach for tele-rehabilitation based on deep learning 3D human pose estimation. Our approach aims to evaluate the effectiveness and practicality of the tele-rehabilitation method over a 12-week experiment. Building upon previous research, our hypothesis is that 3D pose estimation is equally as effective as traditional rehabilitation methods in enhancing muscle quality, strength, and functionality in older adults with sarcopenia.

## Methods

This study is a single-center, prospective, unmasked (with randomization concealment) randomised controlled trial introducing a hybrid comprehensive tele-rehabilitation program in elder patients with sarcopenia, conducted between May to August 2023. The trial was approved by the Ethics Committee of Northeast Normal University (number: NC2022121703) and registered in ClinicalTrials.gov (NCT05767710)(14/03/2023). Participants signed an informed consent upon their agreement to participate in the study. We conducted the study at the Chinese Research Center for Exercise Epidemiology.

### Participants and randomization

To determine the appropriate sample size for our study, we used G*Power for statistical power analysis [34]. We based on Cohen’s-D formulas, set the effect size to 0.4, the power to 90%, the α error probability to 0.05, the number of groups to 3, and the number of measurements to 3. Based on these settings, GPower calculated that we needed at least 17 participants in each group to achieve sufficient statistical power. To account for possible dropouts, we increased the sample size by 20%, resulting in a minimum of 21 participants per group. Since we had three groups in our study, the total sample size required was at least 63 individuals.

Considering that the prevalence of sarcopenia among older adults in China is about 17% [35], and combined with the minimum number of experiments needed for this study, we recruited 384 volunteers from six communities in Changchun, China, using flyers, social media, and word-of-mouth. Finally, We recruited 75 participants in this experiment according to the following inclusion and exclusion criteria. Inclusion criteria: (1) aged beteween 60–75 years; (2) meet the screening criteria for sarcopenia in Asia established by AWGS 2019 [3], details in following part Assessment of the sarcopenia; (3)have a computer at home and (4) who can use a computer or was accompanied by family number who can use a computer. Exclusion criteria: (1) participants taking medications that significantly impact musculoskeletal function; (2) participants suffering from respiratory failure or other bodily problems; (3) participants with mental disorders or neurological disorders; (4) patients who participate in other training programs on a regular basis; (5) have studied Taichi systematically or mastered Taichi proficiently. Stratified randomization was performed based on the gender of the 75 participants, resulting in two groups: males and females. Then, the computer randomization method was employed to generate a random number for each participant, and they were arranged in ascending order according to these random numbers. With the random number sequence as the foundation, the participants were allocated to different groups in a sequential and random manner, face-to-face traditional training group (TRHG), a general remote training group (GTHG), and an AI-based remote training group (AITHG). To ensure rigor, the Taichi videos used in the GTHG and AITHG were recorded by the same Taichi instructor who also provided in-person guidance to the TRHG. The Taichi instructor was only involved in the online video recording and offline course guidance and did not participate in any other experimental procedures.

### Study design

**Fig. 1:**
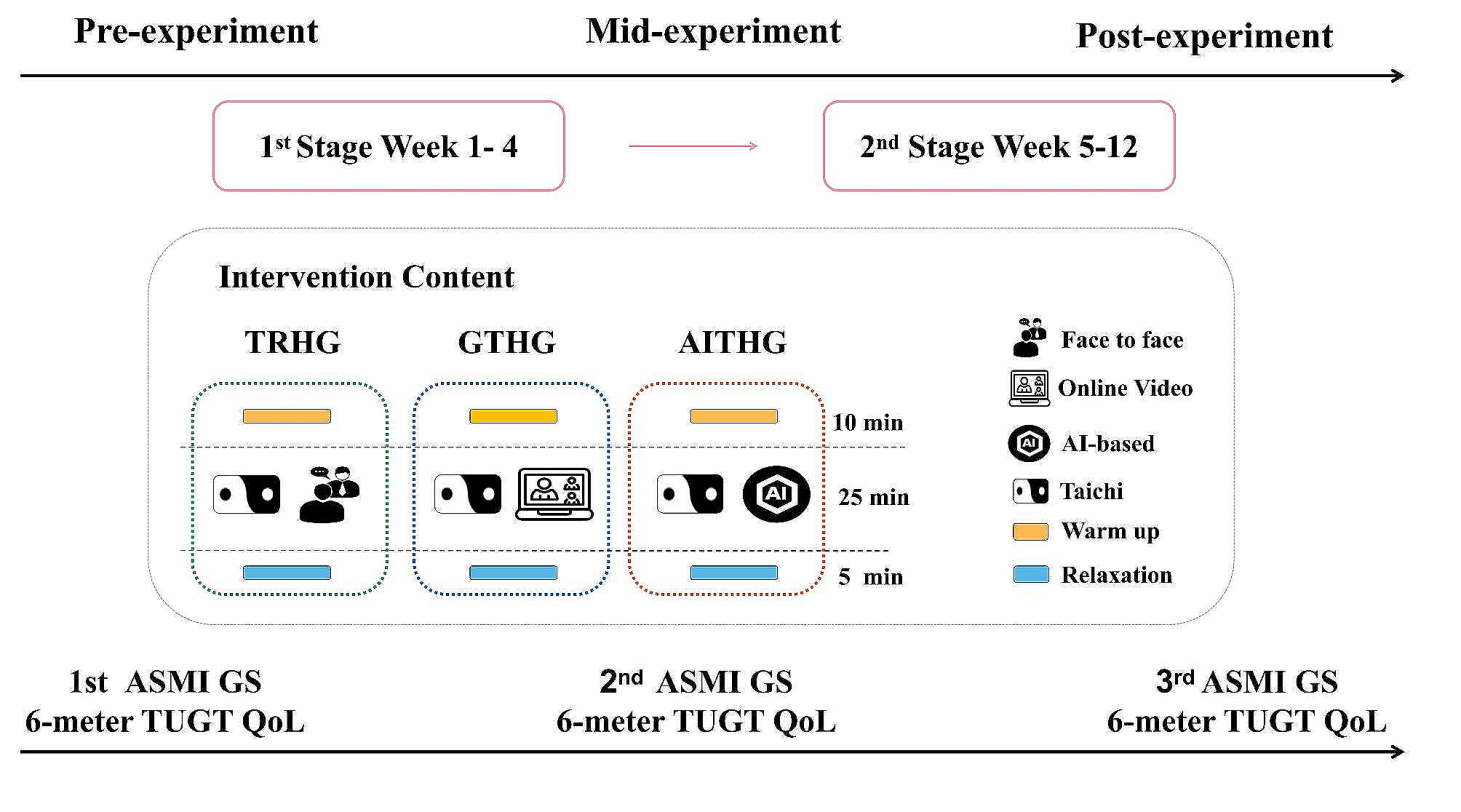
The overall design of the experiment

All intervention groups underwent a 3-month program consisting of 24-form Taichi exercises, with a frequency of three sessions per week. The intervention period can be divided into two stages: Stage 1: Basic Stage (1–4 weeks): This stage is the movement learning and review and consolidation stage, four new movements are learned in each class, the review and consolidation exercises of the first four movements are completed in the second class, and the four new movements are learned in the third class, until all movements are learned and reviewed. Stage 2: Intensive Phase (5–12 weeks): This is the formal practice phase, with two complete sets of Taichi exercises in each lesson. Specifically, the TRHG received a total of 36 in-person sessions of traditional Taichi training. The GTHG utilized Tencent Meeting software for 36 training sessions conducted remotely. Similarly, the AITHG engaged in 36 training sessions using the AI-based remote rehabilitation program proposed in this study. Each session lasting 40 min, with a 10-minute warm-up activity at the beginning and a 5-minute relaxation activity at the end of each session. Prior to the formal experiment, a pre-test was conducted to gather baseline data for all three groups. At the 8-week mark, a critical mid-intervention test was conducted to compare the indices of the participants in each group. This time point was specifically chosen because previous studies have shown that short-term exercise training (< 8 weeks) does not have an effect on muscle structure [36, 37]. Therefore, the 8-week time point offers a pivotal moment to assess the initial benefits of the intervention, providing a more comprehensive evaluation of the rehabilitation effects and intervention efficacy. Following the completion of the experiment, a final post-test was conducted on the participants. The testing content, procedures, sequence, and location were identical to the pre-test, as shown in Fig. 1.

The specific implementation details for each group are as follows:

1) **TRHG**: All sessions were conducted offline and implemented by professional Taichi instructors. The audio from the video used for the other two groups was utilized to assist the practice of participants in TRHG.

2) **GTHG**: All sessions were conducted in an online format, with participants practicing along with Taichi videos while a professional Taichi instructor supervised and guided the movements throughout the session. A computer connected to a high-resolution webcam was used to establish real-time remote visual communication between the rehabilitation unit and the patient’s home via Tencent conferencing software (Meeting-Tencent).

3) **AITHG**: All sessions were conducted online, with participants practicing along with Taichi videos while the AI guided the movements throughout the session. A remote rehabilitation program was installed in patients’ homes by study team members. Participants who do not know how to use a computer were assisted by their family members.

### Assessment of the sarcopenia

Asian Working Group for sarcopenia (AWGS) 2014 consensus defined sarcopenia as age related loss of muscle mass, plus low muscle strength, and/or low physical performance [38]. AWGS 2019 retains the previous definition of sarcopenia but revises some criteria [3]. Therefore, this study used the definition of sarcopenia and chose the latest 2019 criteria as following:

1. **Appendicular skeletal muscle mass index (ASMI)** Inbody S10 Biospace (Biospace Co. Ltd., Korea.) were used. Subjects followed instructions and completed the test. The critical point was the men less than 7.0 kg/m^2^ and women less than 5.7 kg/m^2^.
2. **Muscle strength (grip strength)** Jamar Hydraulic Hand Dynamometer (SH5001, Saehan Corp, Masan, Korea, 2017) were used. Participants were instructed to maintain a natural standing posture during the duration of the test, keeping their wrists in a neutral position and their elbows completely extended. The maximum value was retained after they were given two separate grip strength tests. The critical point was the men less than 28 kg and women less than 18 kg.
3. **Physical performance (6-meter walking pace)** Stopwatch, ruler, tape were used. Subjects were prepared behind the starting line, and when they heard the ”start” command, they walked toward the finish line at a normal speed without accelerating or decelerating, and were measured at least twice to record the average speed. The critical point was less than 1.0 m/s.

### Assessment indicators

#### Measurement method of appendicular skeletal muscle mass index

Appendicular skeletal muscle mass index ( kg/m^2^), which is represented as muscle mass per m^2^ of the limbs, can be used to evaluate sarcopenia [39]. Thus, ASMI was chosen as the main result indicator in the research and was evaluated using multifrequency bioelectrical impedance analysis (Inbody S10 Biospace, Biospace Co. Ltd., Korea).

#### Measurement method of grip strength

Grip strength is one of the most important criteria for diagnosing sarcopenia and a common indirect measure of total muscle strength [3]. Therefore, grip strength was selected as the primary observation and measured it before and after the intervention. We used a Jamar Hydraulic Hand Dynamometer (SH5001, Saehan Corp, Masan, Korea, 2017) to measure grip strength of participants. Participants were instructed to maintain a natural standing posture during the duration of the test. They were also instructed to keep their wrists in a neutral position and their elbows completely extended. The maximum value was retained after they were given two separate grip strength tests.

#### Measurement method of 6-meter walking pace

In epidemiologic studies, walking speed is an important measure of functioning that strongly shows performance-based measures of lower body function and predicts mortality [40]. 6-meter walking pace is also one of the most important criteria for diagnosing sarcopenia [3]. Participants positioned themselves behind the starting line and, upon hearing the instruction “begin,” proceeded steadily towards the endpoint six meters away. Timing was concluded upon reaching the endpoint without any acceleration or deceleration during the course. The measurement was repeated twice, and the average velocity was recorded in seconds, with precision extended to two significant figures beyond the decimal point.

#### Measurement method of timed-up-and-go test

The research indicates that the Timed-Up-and-Go Test (TUGT) can reflect the health status, daily activity capacity, fall risk and fear of falling [41] in the older adults population. Furthermore, there is a significant correlation between the performance of the TUGT and the rate of all-cause mortality [42]. Given that the testing procedure of TUGT involves “passing a marker three meters away and turning back to the starting position” and requires participants to complete the task “as quickly as possible,” it simulates real-life situations that involve rapid movements and agile turning. This aspect allows for the evaluation of stability and control capabilities during rapid movements and turns. Participants were seated in an upright position on a chair, with their feet placed flat on the ground, ensuring a stable posture. The experimenter initiated the test by delivering the verbal cue “begin” while simultaneously commencing the stopwatch. From a seated position, participants volitionally rose, circumnavigated a marker positioned three meters away, executed a rapid about-face maneuver, and proceeded to return to the original seated posture, whereupon the timing ceased. Participants were instructed to complete the task expeditiously while maintaining utmost caution to ensure personal safety.

#### Measurement method of QoL

Research findings indicate that individuals with sarcopenia, on average, have a lower QoL (QoL) [43]. The SF-36 questionnaire encompasses eight distinct health scales, namely physical functioning (10 items), role limitations–physical (4 items), bodily pain (2 items), general health (5 items), vitality (4 items), social functioning (2 items), role limitations–emotional (3 items), and mental health (5 items). From these eight scales, two fundamental dimensions of health can be derived: the physical dimension and the mental dimension. Additionally, there is an individual item, separate from the aforementioned scales, dedicated to assessing any noticeable changes in health. The SF-36 is suitable for use with older adults [44].

### Tele-rehabilitation program design

#### 3D human pose estimation model

Real-time feedback and correction of incorrect movements during Taichi practice are crucial for providing effective remote rehabilitation guidance. In this study, we adopted MediaPipe, a framework developed by Google for building multimodal audio, video, or any other time series data [45]. MediaPipe is a versatile, cross-platform tool that is easy to install on personal computers. The MediaPipe Pose module features BlazePose [46], which is a highly optimized skeletal tracking algorithm for real-time 3D pose estimation from live video streams. This model can achieve at least 30 frames per second on CPU-only devices. It has been trained and optimized for high accuracy in extracting 33 skeletal keypoints, which include shoulder peaks, elbow joints, wrist joints, hip joints, knee joints, and ankle joints. This enables precise tracking of Taichi movements, especially for those who require accurate postures.

#### 3D coordinate extraction of skeletal keypoints

BlazePose is a top-down prediction network structure that utilizes the correlation between poses and bounding boxes across frames to accurately predict keypoints. To extract the 3D coordinates of skeletal keypoints, the first step involves using the OpenCV library to read a video of a Taichi demonstration. Frame by frame, these images are inputted into the BlazePose model to identify the coordinates of various skeletal keypoints in 3D space for the Taichi instructor. This process includes an encoder for extracting features from the images and a decoder for regressing the coordinates. The model accurately identifies the 33 skeletal keypoints, including shoulder peaks, elbow joints, wrist joints, hip joints, knee joints, and ankle joints, in each frame of the video and outputs their corresponding 3D coordinates.

To ensure smooth trajectories of the Taichi instructor’s movements, the 3D coordinates of all keypoints obtained in each frame undergo a smoothing process. This helps eliminate potential noise and jitter, ensuring a stable trajectory for subsequent motion analysis. The smoothness of the trajectory ensures a reliable foundation of data for further analysis of Taichi movements.

#### Taichi movement feature extraction

After using the BlazePose model to extract key points frame by frame from a demonstration video of a Taichi instructor’s movements, data containing 3D coordinates for 33 skeletal keypoints was obtained. However, following consultation with a Taichi instructor and two medical professionals, 15 of these keypoints, including 10 facial and 6 hand keypoints, were deemed unimportant or unnecessary and were thus removed. The final 17 keypoints, including the nose as a reference point for determining the relative positioning between the head and torso, were selected specifically for use in the extraction of Taichi features. Furthermore, we determined the midpoint of the line connecting two points based on the position of the left and right hip joints and used it as the center of gravity to indicate the change of center of gravity in Taichi movements. To provide precise guidance for each Taichi movement, the system segmented Taichi videos and distinguished different actions according to time settings. With guidance from an experienced Taichi coach, the system extracted the main features of each movement and provided real-time feedback to participants on the necessary improvements for each movement based on the relative positions of skeletal key points and the angles between joints, which can be obtained through the cosine rule. Finally, we deployed the model on a local computer, configured it as an accessible server via intranet penetration technology, and opened it up to all participants in the AITHG, enabling the model to capture the movement trajectories of participants while protecting their privacy and ensuring their participation in Taichi training according to the established schedule.

#### Model corretction

The remote rehabilitation model analyzes users’ movements using machine learning algorithms and compares them with standard movements to determine their accuracy. If the model detects any deviation in users’ movements, it issues a prompt and explains in detail where the deviation occurred in the specific body part and how to correct it. Users can determine the accuracy of their movements and correct their errors through real-time feedback and textual prompts. For example, when a user deviates or performs an incorrect movement, the model prompts and identifies the significant deviation, such as “the right knee joint is bent at too large of an angle” or “the left elbow joint is bent at too small of an angle.” This real-time feedback helps users correct their movements in a timely manner, avoiding adverse effects and improving their understanding and mastery of rehabilitation training techniques, ultimately enhancing the effectiveness of their rehabilitation training.

### Mathematical statistics

The data in this study were processed and analyzed using SPSS 26.0. All data in this study were continuous variables with no outliers. Therefore, the mean and standard deviation (Mean ± SD) were used to represent the data. Single-factor analysis of variance (ANOVA) and repeated measures ANOVA were used to compare the differences in central tendency among the three groups before and after the experiment. The Kolmogorov-Smirnov (K-S) test was used to assess the normality of all continuous variables. If the data did not follow a normal distribution, logarithmic transformation was applied to normalize the distribution. When the data did not meet the assumption of sphericity according to the Mauchly’s sphericity test, the results of the corrected Pillai’s Trace in multivariate analysis of variance (MANOVA) were used. In this study, the significance level was represented by *p*, and *p* < 0.05 was defined as a significant difference, while *p* < 0.01 was defined as a highly significant difference.

## Results

**Fig. 2:**
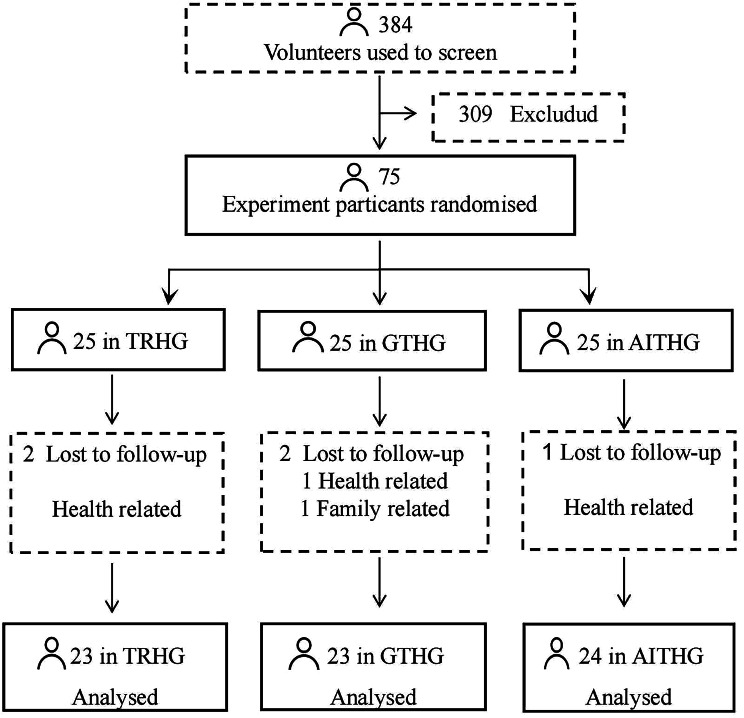
The participants of the experiment

According to the study inclusion and exclusion criteria, a total of 75 eligible participants were screened in this study and randomly divided into three groups: TRHG, GTHG and AITHG. In the course of the experiment, two subjects in each of the TRHG and GTHG withdrew halfway due to physical or family reasons, and one in the AITHG withdrew. Finally, a total of 70 subjects completed the entire exercise intervention process, including 23 in the TRHG, 23 in the GTHG, and 24 in the AITHG, as shown in Fig. 2.

### Experimental feasibility assessment

The basic information of participants in this study are shown in Table 1. There is no significant difference between the three groups of subjects in terms of the basic conditions of age, height, weight and BMI, and there is no significant difference in the results of the performance of the five indicators, which indicates the feasibility of the experiment.

**Table 1:** Basic information of participants

Items	TRHG (*n* = 23)	GTHG (*n* = 23)	AITHG (*n* = 24)	*p*
Age (years old)	70.91 ± 3.94	72.26 ± 4.43	73.67 ± 4.77	0.108
Height (cm)	159.60 ± 5.42	160.21 ± 5.11	158.77 ± 6.09	0.673
Weight (kg)	58.35 ± 5.90	61.22 ± 6.06	60.22 ± 5.52	0.244
BMI(kg/m2 )	22.93 ± 2.37	23.86 ± 2.17	23.89 ± 1.78	0.225
ASMI(kg/m2 )	4.82 ± 0.80	4.53 ± 0.81	4.79 ± 0.79	0.407
Grip Strength (kg)	21.64 ± 3.49	19.69 ± 3.51	20.74 ± 3.40	0.171
6-meter walking pace(m/s)	0.65 ± 0.11	0.63 ± 0.13	0.64 ± 0.13	0.877
TUGT (s)	13.91 ± 0.67	13.69 ± 0.70	13.79 ± 0.85	0.603
QoL (scores)	66.03 ± 7.71	63.68 ± 7.02	65.65 ± 5.24	0.449

This table presents basic information of participants

### Comparison of ASMI results in three groups

As shown in Table 2, the interaction effect between the number of measurements and group was significant in the comparison of the test results of ASMI among the three groups of subjects, F = 6.219, *p* < 0.01, partial *η*^*2*^ = 0.157. Intragroup results revealed a highly significant difference in TRHG wherein participants exhibited a statistically significant increase in ASMI by 0.169 kg/m^2^ (*95% CI*: 0.073, 0.265) after the 8th week of the intervention compared to baseline. Following the 12-week experiment, ASMI significantly increased by 0.327 kg/m^2^ (*95% CI*: 0.176, 0.479) (*p* < 0.01) compared to baseline, and showed a highly significant improvement of 0.158 kg/m^2^ (*95% CI*: 0.060, 0.257) (*p* < 0.01) compared to the mid-experiment ASMI. On the other hand, in GTHG, the difference in ASMI mean compared to baseline after the 8th week of the experiment was not statistically significant (*p* > 0.05), with a mean difference of -0.073 kg/m^2^ (*95% CI*: -0.170, 0.23). However, after the 12-week experiment, ASMI significantly increased by 0.179 kg/m^2^ (*95% CI*: 0.027, 0.330) (*p* < 0.05) compared to baseline, and showed a highly significant improvement of 0.252 kg/m^2^ (*95% CI*: 0.154, 0.351) (*p* < 0.01) compared to the mid-experiment ASMI. Similarly, in AITHG, there was a highly significant difference as participants exhibited a statistically significant increase in ASMI by 0.132 kg/m^2^ (*95% CI*: 0.038, 0.226) after the 8th week of the experiment compared to baseline. Following the 12-week intervention, ASMI significantly increased by 0.367 kg/m^2^ (*95% CI*: 0.218, 0.515) (*p* < 0.01) compared to baseline, and showed a highly significant improvement of 0.235 kg/m^2^ (*95% CI*: 0.138, 0.331) (*p* < 0.01) compared to the mid-experiment ASMI.

Inter-group results indicate a significant difference in the mid-experiment at the 8th week, with the order of ASMI mean values as follows: TRHG > AITHG > GTHG. The difference in ASMI levels between participants in the TRHG and AITHG was 0.065 (*95% CI*: -0.481, 0.611) kg/m^2^with no statistically significant difference (*p* > 0.05). The difference in ASMI levels between participants in the TRHG and GTHG was 0.530 (*95% CI*: -0.022, 1.082) kg/m^2^, with no statistically significant difference (*p* > 0.05). The difference in ASMI levels between participants in the AITHG and GTHG was 0.465 (*95% CI*: -0.081, 1.011) kg/m^2^, with no statistically significant difference (*p* > 0.05). At the end of the 12-week intervention, the late-experiment comparison of ASMI mean values showed the order as follows: AITHG > TRHG > GTHG. The difference in ASMI levels between participants in the TRHG and AITHG was − 0.011 (*95% CI*: -0.559, 0.536) kg/m^2^, with no statistically significant difference (*p* > 0.05). The difference in ASMI levels between participants in the TRHG and GTHG was 0.436 (*95% CI*: -0.117, 0.989) kg/m^2^, with no statistically significant difference (*p* > 0.05). The difference in ASMI levels between participants in the AITHG and GTHG was 0.448 (*95% CI*: -0.100, 0.995) kg/m^2^, with no statistically significant difference (*p* > 0.05).

**Table 2:** Comparison of ASMI results in three groups (kg/m^2^ )

Items	TRHG (*n* = 23)	GTHG (*n* = 23)	AITHG (*n* = 24)
pre-experiment	4.82 ± 0.80	4.53 ± 0.81	4.79 ± 0.79
mid-experiment	4.99 ± 0.72^aa^	4.46 ± 0.78	4.92 ± 0.79^aa^
post-experiment	5.14 ± 0.68^bbcc^	4.71 ± 0.81^bcc^	5.16 ± 0.80^bbcc^

This table presents comparison of ASMI results in three groups.^a^ Indicates a statistically significant difference within the group at mid-experiment compared to pre-experiment; ^aa^ Indicates a highly significant difference within the group in the mid-experiment compared to the pre-experiment ; ^b^ Indicates that there is a statistical difference within the group in the post-experiment than in the pre-experiment; ^bb^ Indicates that there is a highly significant difference within the group in the post-experiment than in the pre-experiment ; ^c^ Indicates that there is a statistical difference within the group in the post-experiment than in the mid-experiment; ^cc^ Indicates that there is a highly significant statistical difference within the group in the post- experiment than in the mid-experiment

### Comparison of grip strength results in three groups

As shown in Table 3, the interaction effect between the number of measurements and groups in the comparison of grip strength test results among the three groups of participants was not statistically significant, with an F value of 0.537 (*p* > 0.05) and a partial *η*^*2*^ of 0.016. Within-group results revealed that for the TRHG, the mean difference in grip strength level after 8 weeks of experiment compared to pre-experiment was − 0.176 (*95% CI*: -0.596, 0.243) kg, but there was no statistically significant difference (*p* > 0.05). The mean difference in grip strength level after 12 weeks of experiment compared to pre-experiment was − 0.204(*95% CI*: -0.857, 0.448) kg, again without a statistically significant difference (*p* > 0.05), and compared to mid-experiment grip strength, the mean difference was − 0.028 (*95% CI*: -0.441, 0.385) kg, which was not statistically significant (*p* > 0.05). For the GTHG, the mean difference in grip strength level after 8 weeks of experiment compared to pre-experiment was − 0.317 (*95% CI*: -0.736, 0.102) kg, without a statistically significant difference (*p* > 0.05). After 12 weeks of experiment, the mean difference in grip strength level compared to pre-experiment was − 0.474 (*95% CI*: -1.127, 0.178) kg, still without a statistically significant difference (*p* > 0.05). Compared to mid-experiment grip strength, the mean difference was − 0.157 (*95% CI*: -0.571, 0.256) kg, again, not statistically significant (*p* > 0.05). For the AITHG, the mean difference in grip strength level after 8 weeks of experiment compared to pre-experiment increased by 0.013 (*95% CI*: -0.398, 0.423) kg, but there was no statistically significant difference (*p* > 0.05). The mean difference in grip strength level after 12 weeks of experiment compared to pre-experiment was − 0.080(*95% CI*: -0.719, 0.559)kg, without a statistically significant difference (*p* > 0.05), and compared to mid-experiment grip strength, the mean difference was − 0.093 (*95% CI*: -0.497, 0.312) kg, also not statistically significant (*p* > 0.05).

Inter-group results indicate that, after the 8th week of the experiment, the mean grip strength comparison between groups revealed that TRHG > AITHG > GTHG. The observed difference in grip strength between the TRHG and the AITHG was 0.716 (*95% CI*: -1.636, 3.068)kg, and this difference was not statistically significant (*p* > 0.05). Similarly, the observed difference in grip strength between the TRHG and the GTHG was 2.087(*95% CI*: -0.290, 4.464)kg, and this difference was not statistically significant (*p* > 0.05). Additionally, no statistically significant difference (*p* > 0.05) was found in grip strength between the AITHG and the GTHG, with an observed difference of 1.371(*95% CI*: -0.981, 3.723) kg. At the end of the 12th week, the mean grip strength comparison between groups showed that TRHG > AITHG > GTHG. The observed difference in grip strength between the TRHG and the AITHG was 0.780 (*95% CI*: -1.462, 3.023) kg, and this difference was not statistically significant (*p* > 0.05). The observed difference in grip strength between the TRHG and the GTHG was 2.216 (*95% CI*: -0.050, 4.482) kg, and this difference was not statistically significant (*p* > 0.05). Additionally, no statistically significant difference (*p* > 0.05) was found in grip strength between the AITHG and the GTHG, with an observed difference of 1.436(*95% CI*: -0.807, 3.678) kg.

**Table 3:** Comparison of grip strength results in three groups (kg)

Items	TRHG (*n* = 23)	GTHG (*n* = 23)	AITHG (*n* = 24)
pre-experiment	21.64 ± 3.49	19.69 ± 3.51	20.74 ± 3.40
mid-experiment	21.46 ± 3.12	19.38 ± 3.38	20.75 ± 3.36
post-experiment	21.44 ± 2.83	19.22 ± 3.29	20.66 ± 3.26

This table presents comparison of grip strength results in three groups

### Comparison of 6-meter walking pace results in three groups

As shown in Table 4, the comparison of measurement times and group effects in the test results of the 6-meter walking pace among the three groups showed no significant experiment effect (F = 0.017, *p* > 0.05, partial *η*^*2*^ = 0.001). Within-group results showed that after 8 weeks of experiment, the 6-meter walking pace of participants in the TRHG group highly significant increased by 0.096 (*95% CI* : 0.023, 0.169) m/s compared to before the experiment (*p* < 0.01). After 12 weeks of experiment, the 6-meter walking pace significantly increased by 0.190 (*95% CI* : 0.085, 0.294) m/s compared to before the experiment (*p* < 0.01), and it was also significantly higher by 0.093 (*95% CI* : 0.022, 0.165) m/s compared to the mid-experiment 6-meter walking pace during the experiment (*p* < 0.01). In the GTHG, after 8 weeks of experiment, the 6-meter walking pace achieved a highly significant difference by 0.098 (*95% CI* : 0.025, 0.171) m/s compared to before the experiment (*p* < 0.01). After 12 weeks of experiment, the 6- meter walking pace highly significantly increased by 0.189 (*95% CI* : 0.085, 0.294) m/s compared to before the experiment (*p* < 0.01), and it was also highly significantly by 0.091 (*95% CI* : 0.020, 0.163) m/s compared to the mid-experiment 6-meter walking pace during the experiment (*p* < 0.01). In the AITHG, after 8 weeks of intervention, the 6-meter walking pace highly significantly increased by 0.105 (*95% CI* : 0.034, 0.177) m/s compared to before the experiment (*p* < 0.01). After 12 weeks of experiment, the 6- meter walking pace highly significantly increased by 0.195 (*95% CI* : 0.093, 0.297) m/s compared to before the experiment (*p* < 0.01), and it was also highly significantly by 0.090 (*95% CI* : 0.019, 0.160) m/s compared to the mid-experiment 6-meter walking pace during the experiment (*p* < 0.01).

The inter-group results show that, after the 8th week of the experiment, the comparison of 6-meter walking pace levels is TRHG > AITHG > GTHG. The difference in 6-meter walking pace levels between the TRHG and the AITHG is 0.005 m/s (*95% CI*: -0.141, 0.152), and there is no statistically significant difference (*p* > 0.05). The difference in 6-meter walking pace levels between the TRHG and the GTHG is 0.016 m/s (*95% CI*: -0.133, 0.164), and there is no statistically significant difference (*p* > 0.05). The difference in 6-meter walking pace levels between the AITHG and the GTHG is 0.010 m/s (*95% CI*: -0.136, 0.157), and there is no statistically significant difference (*p* > 0.05). At the end of the 12th week of intervention, the comparison of 6-meter walking pace levels in the later stage of the experiment is TRHG > AITHG > GTHG. The difference in 6-meter walking pace levels between the TRHG and the AITHG is 0.009 m/s (*95% CI*: -0.165, 0.183), and there is no statistically significant difference (*p* > 0.05). The difference in 6-meter walking pace levels between the TRHG and the GTHG is 0.018 m/s (*95% CI*: -0.158, 0.194), and there is no statistically significant difference (*p* > 0.05). The difference in 6-meter walking pace levels between the AITHG and the GTHG is 0.009 m/s (*95% CI*: -0.166, 0.183), and there is no statistically significant difference (*p* > 0.05).

**Table 4:** Comparison of 6-meter walking pace results in three groups (m/s)

Items	TRHG (*n* = 23)	GTHG (*n* = 23)	AITHG (*n* = 24)
pre-experiment	0.65 ± 0.11	0.63 ± 0.13	0.64 ± 0.13
mid-experiment	0.75 ± 0.18^aa^	0.73 ± 0.22^aa^	0.74 ± 0.21^aa^
post-experiment	0.84 ± 0.27^bbcc^	0.82 ± 0.24^bbcc^	0.83 ± 0.22^bbcc^

This table presents comparison of 6-meter walking pace results in three groups

### Comparison of TUGT results in three groups

As shown in Table 5, the intergroup results showed that the interaction effect of measurement times and groups in the TUGT timing test was not significant (F = 0.107, *p* > 0.05, *η*^*2*^ = 0.003). Within-group results showed that in the TRHG, the TUGT test timing significantly improved by 0.444 (*95% CI*: -0.823, -0.064) s, (*p* < 0.05) in the mid-term of the experiment compared to pre-experiment. After 12 weeks of experiment, the TUGT test timing highly significantly improved by 1.00 (*95% CI*: -1.534, -0.468) s, (*p* < 0.01) compared to that of pre-experiment, and highly significantly improved by 0.557 (*95% CI*: -0.978, -0.136) s, (*p* < 0.01), compared to the mid-experiment. In the GTHG, the TUGT test timing improved by 0.329 (95% CI: -0.709, -0.050) s, (*p* > 0.05), after the 8th week of the mid-term compared to before the experiment. After 12 weeks of experiment, the TUGT test timing highly significantly improved by 0.831 (*95% CI*: -1.364, -0.298) s, (*p* < 0.01) compared to before the experiment, and significantly improved by 0.502 (*95% CI*: -0.923, -0.081) s, (*p* < 0.05) compared to the mid-term of the experiment. In the AITHG, the TUGT test timing significantly improved by 0.418 (*95% CI*: -0.790, -0.047) s, (*p* < 0.05), after the 8th week of the mid-term compared to before the experiment. After 12 weeks of experiment, the TUGT test timing highly significantly improved by 0.986 (*95% CI*: -1.508, -0.465) s, (*p* < 0.01), compared to before the experiment, and highly significantly improved by 0.568 (*95% CI*: -0.980, -0.156) s, (*p* < 0.01), compared to the mid-term of the experiment.

The intergroup results showed that in the mid-experiment, the TUGT timing test result is GTHG < AITHG < TRHG. The difference in TUGT timing test between TRHG and AITHG was 0.101(*95% CI*: -0.671, 0.873) s, and there was no statistically significant difference (*p* > 0.05); the difference in TUGT timing test between TRHG and GTHG was 0.107 (*95% CI*: -0.674, 0.887)s, and there was no statistically significant difference (*p* > 0.05); the difference in TUGT timing test between AITHG and GTHG was 0.006(*95% CI*: -0.766, 0.778)s, and there was no statistically significant difference (*p* > 0.05). At the end of the intervention, the TUGT timing test comparison results is AITHG < GTHG < TRHG. The difference in TUGT timing test between TRHG and AITHG was 0.111 (*95% CI*: -0.771, 0.994)s, and there was no statistically significant difference (*p* > 0.05); the difference in TUGT timing test between TRHG and GTHG was 0.051 (*95% CI*: -0.841, 0.943)s, and there was no statistically significant difference (*p* > 0.05); the difference in TUGT timing test between AITHG and GTHG was − 0.061 (*95% CI*: -0.943, 0.822)s, and there was no statistically significant difference (*p* > 0.05).

**Table 5:** Comparison of TUGT results in three groups (s)

Items	TRHG (*n* = 23)	GTHG (*n* = 23)	AITHG (*n* = 24)
pre-experiment	13.91 ± 0.67	13.69 ± 0.70	13.79 ± 0.85
mid-experiment	13.47 ± 0.96^a^	13.36 ± 1.06	13.37 ± 1.20^a^
post-experiment	12.91 ± 1.30^bbcc^	12.86 ± 1.11^bbc^	12.80 ± 1.29^bbcc^

This table presents comparison of TUGT results in three groups

### Comparison of QoL results in three groups

As shown in Table 6, there was no significant interaction effect between the measurement times and groups in the comparison of SF-36 QoL scores among the three groups, F = 0.304, *p* > 0.05, partial *η*^*2*^ = 0.009. Within-group results showed that in the TRHG, the SF-36 QoL scores of the participants in the TRHG highly significantly increased by 3.205 (*95% CI*: 1.121, 5.289) (*p* < 0.01) of the 8th week compared to before the experiment. The SF-36 scores at the 12th week intervention were higher than those before the experiment, with a highly significant difference of 3.689 (*95% CI*: 1.540, 5.839) (*p* < 0.01). Moreover, the SF-36 scores at 12th week were significantly higher than those at the mid-experiment, with a significant difference of 0.484 (*95% CI*: 0.064, 0.905) (*p* < 0.05). In the GTHG, the SF-36 scores at the 8th week and 12th week intervention were highly significantly increased compared to those before the experiment, with a highly significant difference of 2.910 (*95% CI*: 0.826, 4.995) (*p <* 0.01) and 3.297 (*95% CI*: 1.148, 5.447) (*p* < 0.01), respectively. In addition, the SF-36 scores at 12th week were significantly higher than those at the mid-experiment, but with a non-significant difference of 0.387 (*95% CI*: -0.034, 0.808) (*p* > 0.05). For the AITHG, the SF-36 scores at late phase of the 8th week and 12th week intervention were highly significantly increased compared to those before the experiment, with a highly significant difference of 4.000 (*95% CI*: 1.960, 6.040) (*p* < 0.01) and 4.361 (*95% CI*: 2.257, 6.465) (*p* < 0.01), respectively. Moreover, the SF-36 scores at 12th week were slightly higher than those at the middle phase of the experiment, with a non-significant difference of 0.361 (*95% CI*: -0.051, 0.773) (*p* > 0.05).

The intergroup results showed that at the mid-experiment, the comparison of SF-36 QoL scores was as follows: AITHG > TRHG > GTHG. The difference in SF-36 scores between the TRHG and the AITHG was − 0.419 (*95% CI*: -6.241, 5.403), indicating no significant difference (*p* > 0.05). The difference in SF-36 scores between the TRHG and the GTHG was 2.639 (*95% CI*: -3.245, 8.523), also indicating no significant difference (*p* > 0.05). The difference in SF-36 scores between the AITHG and the GTHG was 3.058 (*95% CI*: -2.764, 8.880), again indicating no significant difference (*p* > 0.05). At the end of the 12th week intervention, the comparison of SF-36 health-related QoL scores was as follows: AITHG > TRHG > GTHG. The difference in SF-36 scores between the TRHG and the AITHG was − 0.295 (*95% CI*: -6.618, 5.577), indicating no significant difference (*p* > 0.05). The difference in SF-36 scores between the TRHG and the GTHG was 2.737 (*95% CI*: -3.199, 8.672), also indicating no significant difference (*p* > 0.05). The difference in SF-36 scores between the AITHG and the GTHG was 3.032 (*95% CI*: -2.841, 8.905), once again indicating no significant difference (*p* > 0.05).

**Table 6:** Comparison of QoL results in three groups (scores)

Items	TRHG (*n* = 23)	GTHG (*n* = 23)	AITHG (*n* = 24)
pre-experiment	66.03 ± 7.71	63.68 ± 7.02	65.65 ± 5.24
mid-experiment	69.23 ± 9.32^aa^	66.60 ± 7.91^aa^	69.65 ± 7.11^aa^
post-experiment	69.72 ± 9.35^bbc^	66.98 ± 7.96^bb^	70.01 ± 7.24^bb^

This table presents comparison of QoL results in three groups

## Discussion

### The effects of three training methods on muscle mass indicators in older adults with sarcopenia

The findings of this experiment reveal that after a 3-month intervention, consisting of 24-form Taichi practice three times per week for 40 min per session, there were extremely significant improvements in the mid-experiment compared to the pre- experiment, post-experiment compared to the pre-experiment, and post-experiment compared to the mid-experiment results in terms of the mean ASMI in the TRHG. In the GTHG, there was no statistically significant improvement in the mid-experiment compared to the pre-experiment, but a significant improvement was observed in the post- experiment compared to the pre-experiment, and a highly significant improvement in the post-experiment compared to the mid-experiment ASMI. Similarly, in the AITHG, there were highly significant improvements in the mid-experiment compared to the pre-experiment, post-experiment compared to the pre-experiment, and post-experiment compared to the mid-experiment ASMI, providing evidence that Taichi exercise, as a low-intensity aerobic activity, can improve muscle quality in patients with muscular atrophy. The study [47] found that Brain-derived neurotrophic factor (BDNF) is a neurotrophin associated with neuronal growth, differentiation, and plasticity. BDNF is produced in skeletal muscle cells especially during muscle contractions, acting in an autocrine or paracrine manner, and playing an important role in muscle repair. Our findings are also consistent with multiple research studies [48, 49].

The inter-group comparisons of the research results revealed no statistically significant differences in both the mid-term and post-term pairwise comparisons, indicating that the AI remote training group based on deep learning-based 3D human pose estimation technology proposed in this study achieved comparable results to the traditional face-to-face training group in terms of ASMI improvement. The deep learning-based 3D human pose estimation technology involves two steps in the pose estimation process: first, the detector locates the regions of interest for poses in the images, and second, the tracker estimates the keypoints from the regions of interest to obtain the coordinates of the skeletal keypoints for areas such as the head, neck, shoulders, arms, and legs of the participants. By comparing these coordinate information with pre-input standard template actions, this technology can identify and correct defects and inconsistencies in real-time and accuracy during movements, thus assisting individuals with sarcopenia to complete training actions more precisely. The findings of this study align with previous research, demonstrating that incorrect movement postures during high-powered and vigorous exercises can lead to temporary or permanent disabilities [27]. Ensuring the correctness of postures can ensure proper muscle activation during training, helping participants concentrate weight and load on the target muscles, ensuring ideal stretch and contraction of muscles at appropriate angles, increasing the area of muscle force application, stimulating the involvement of more muscle fibers, avoiding unnecessary tension or excessive dependence on other muscle groups, and promoting improvement in muscle quality. Remote rehabilitation serves as an effective means to ensure posture correctness. Chen and Yang [50] designed an end-to-end computer vision application using pose estimation and machine learning to provide personalized feedback on fitness movement forms. The program model utilizes the output results of pose estimation to evaluate exercise videos based on human pose keypoints, determining the correctness of postures. Bernardo et al. [51] uses human pose estimation techniques to help discern whether the user maintains proper posture during weightlifting for biceps curls, identifying movement errors as elbow instability, trunk sway, or limited range of motion.

In conclusion, practicing Taichi can promote muscle mass growth in older adults with sarcopenia. The AI remote program based on deep learning and 3D human pose estimation technology proposed in this study can assist subjects in activating and stimulating target muscle groups correctly, thereby improving muscle quality.

### The effects of three training methods on muscle strength indices in older adults with sarcopenia

According to the results of this study, all three groups of subjects showed a slight decrease in grip strength in the post-experiment compared to the pre-experiment stage, but none of these changes reached statistical significance. This result is consistent with some previous findings in the literature. Huang et al. [52] concluded that the benefits of Taichi exercise in terms of grip strength improvement were not significant. The lack of significant impact on grip strength improvement by practicing Taichi may be attributed to several factors. Firstly, Taichi training itself may not entail a high intensity of upper limb movement, which could limit its specific effect on grip strength. Additionally, the insufficient duration and low frequency of Taichi practice in this study could have contributed to the observed lack of improvement in grip strength.

In a study examining the effects of Taichi on the lower limbs of practitioners, Bagiartana and Huriah [53] conducted a systematic review and meta-analysis of the evidence regarding the effects of Taichi on balance and lower limb strength in community-dwelling older adults. The results indicated that Taichi exercise can effectively improve both balance and lower limb muscle strength in this population. During Taichi practice, the muscles of the lower limbs are constantly contracting and relaxing, and bearing weight, in a variety of different movements and postures. This continuous and varied engagement of the lower limb muscles may lead to greater muscular strength.

In conclusion, the practice of Taichi has been shown to promote the growth of lower limb muscle strength. However, further in-depth research is required to ascertain whether it can significantly increase upper limb strength. The suboptimal effects on upper limb strength may be attributed to factors such as the frequency, duration, and intensity of Taichi practice.

### The effects of three training methods on physical mobility indicators in older adults with sarcopenia

The intra-group results of the 6-meter walking pace in this study demonstrated highly significant improvements across all three experimental groups when comparing the mid-term to the pre-term, the post-term to the pre-term, and the post-term to the mid-term. Silva et. al. [54] and Lage et al. [55], found that sarcopenia is associated with worse health outcomes in Chronic obstructive pulmonary disease (COPD), which will result in the increase in oxidative stress-related factors and the reduction of respiratory muscle strength. Exercise training improved respiratory muscle strength with concurrent improvement of exercise capacity [56]. Huang [57] revealed in their study that a Taichi training program significantly reduced the neuromuscular response time in the rectus femoris, semitendinosus, gastrocnemius, and tibialis anterior muscles of the lower limb in older adults with sarcopenia. Furthermore, dynamic postural control abilities were significantly enhanced. Millor [58] found a significant correlation between lower limb muscle mass and walking ability following their analysis of functional tests and muscle mass. These studies collectively demonstrate that the movement patterns and requirements of Taichi exercises effectively activate and train the muscles of the lower limb, such as the rectus femoris, semitendinosus, and gastrocnemius, leading to improvements in neuromuscular response speed, walking ability.

Participants in the TRHG group showed a significant increase at mid-experiment compared to pre-experiment, and a highly significant increase at post-experiment compared to pre-experiment and at post-experiment compared to mid-experiment. Participants in the GTHG group showed no statistically significant difference in improvement at mid-experiment compared to pre-experiment, and highlysignificant improvement at post-experiment compared to pre-experiment, and significant improve- ment at post-experiment compared to mid-experiment. The AITHG demonstrated a significant improvement in the mid-term compared to the pre-term, and the post-term showed an highly significant improvement compared to both the pre-term and mid- term. These findings indicate that Taichi exercise has a significant effect in improving physical functional capacity in older adults, with a positive impact on TUGT perfor- mance. A systematic review conducted by Wang [11] gathered a substantial amount of empirical evidence to evaluate the effectiveness of exercise based on traditional Chinese medicine. The quantitative analysis revealed that the Taichi group demonstrated a reduction of 2.62 s in the TUGT compared to the control group. Additionally, the Eight Section Brocade and Qigong exercises resulted in a reduction of 0.96 s and 2 s in the TUGT, respectively. Compared to the other two forms of exercise, Taichi exhibited a favorable advantage in improving TUGT outcomes.

The between-group results for 6-meter walking pace and TUGT in this study showed no statistical difference between the two-by-two comparisons in the middle of the experiment and the two-by-two comparisons in the later part of the experiment. This indicates that the AI remote training group, utilizing the deep learning-based 3D human pose estimation technique proposed in this study, can achieve comparable improvements in 6-meter walking pace and TUGT performance to the traditional face- to-face training group. The remote rehabilitation program utilized by the AITHG can extract motion images of the participants and rapidly analyze their skeletal keypoint information, skeleton information, and motion information through complex algorithms. Traditional clinical assessment methods may be limited in their ability to provide only macroscopic movement characteristics. Pose estimation, as an essential tool for capturing motion information [59], allows for the measurement of fine motor control of the hands and fingers, arm movements, and gait. By extracting detailed movement information, it surpasses the granularity of the Fugl-Meyer scale and enables tailored training and guidance specific to impairments in speed, path curvature, and joint mobility, ultimately enhancing patients’ accuracy and fluidity of movement [60].

In summary, practicing Taichi has the potential to improve physical functioning in older adults with sarcopenia. This study proposes an AI-based remote program utilizing deep learning-based 3D human pose estimation technology to provide precise feedback and guidance by capturing more detailed movement information. Participants can compare their own movements with synchronized reference actions on their terminal devices, identifying deviations and irregularities in their performance and making corrections to ensure accuracy. This continuous training and utilization of the program can contribute to the formation of muscle memory, enabling natural and precise execution of movements, thus enhancing physical activity capabilities.

### The effects of three training methods on QoL indicators in older adults with sarcopenia

According to the within-group results of this study, all three experimental groups demonstrated a significantly improved SF-36 QoL score during the mid-term compared to both the pre-term and post-term assessments. Wang et al. [61] indicate that Taichi exercise can enhance SF-36 QoL scores. This may be attributed to the ability of Taichi to enhance participants’ muscle quality, muscle strength, and physical activity capabilities, subsequently leading to improvements in physiological function and health-related changes in the patients’ QoL scores. The research demonstrates a significant positive correlation between muscle function and QoL, indicating that the better the muscle function, the higher the QoL. Furthermore, in individuals with sarcopenia, measures of muscle strength, such as grip strength [62], and daily physical activity capabilities, such as 6-minute walk speed, are significantly correlated with the overall level of QoL [63]. Additionally, Taichi emphasizes deep and slow breathing, internal meditation, and a state of relaxation. During the practice of Taichi, patients can relax their mind and body, alleviating stress, anxiety, and depression, thereby promoting mental well-being and improving the emotional state and QoL of individuals with sarcopenia. Moreover, the data results of this study show that during the mid-term phase of the experiment, the SF-36 scores of the three groups exhibited a highly significant improvement. After one month of practice, the TRHG showed a significant improvement in the post-term compared to the mid-term, while the AITHG and GTHG did not differ significantly in terms of improvement. This may be due to the fact that factors such as marital status, educational level, income, physical health, and mental well-being, aside from exercise, also influence quality of life. To continuously improve quality of life, it is necessary to consider multiple aspects comprehensively [64].

The SF-36 results of this study indicate that there was no statistically significant difference between the pairwise comparisons of the two groups during the mid-term and post-term experiments. It suggests that the AI remote rehabilitation training group can achieve comparable improvement in the quality of life with traditional rehabilitation methods. This may be because the remote rehabilitation program in the AITHG is not only effective in improving muscle quality and physical activity capabilities, but also offers convenience and flexibility for patients to train at home according to their own schedule, avoiding the inconvenience caused by time and location restrictions such as going out and commuting. This reduces psychological and practical barriers for patients, thus improving their willingness, motivation, and quality of life to participate in training [65, 66].

The limitation of this study is that although the present study achieved the expected research results, it is nonetheless subject to some limitations. On the one hand, the duration of Taichi exercises in this study was limited to 3 months, with 3 sessions per week and each session lasting for 40 min. Despite accruing some amount of training, this duration may not completely reflect the potential effects of long-term exercise on patients with sarcopenia. On the other hand, future research can track the long-term changes in the improvement of sarcopenia in participants in order to better evaluate the persistence and long-term impact of remote training methods using 3D human posture estimation technology based on deep learning.

## Conclusion

Compared to the pre-experiment, there was no significant difference at the post- experiment in the recovery effects on the muscle quality, physical activity ability, and life quality of patients with sarcopenia between the AI-based remote training group and the face-to-face traditional training group. 3D pose estimation is equally as effective as traditional rehabilitation methods in enhancing muscle quality, functionality and life quality in older adults with sarcopenia.

## Data Availability

The datasets generated during and/or analysed during the current study are available from the corresponding author on reasonable request.
